# Preoperative Estimation of Endodontic Working Length with Cone-Beam Computed Tomography and Standardized Paralleling Technique in comparison to Its Real Length

**DOI:** 10.1155/2020/7890127

**Published:** 2020-10-10

**Authors:** Bestoon Mohammed Faraj

**Affiliations:** ^1^Conservative Department, College of Dentistry, University of Sulaimani, Iraq; ^2^Board of Restorative Dentistry, Kurdistan Board of Medical Specialties, Iraq

## Abstract

An accurate estimation of the working canal length is essential for successful root canal treatment. This study is aimed at investigating the diagnostic accuracy of root canal length estimation on cone-beam computed tomography (CBCT) scans and digital paralleling radiographs (PAs), using the real canal length as a gold standard, and at evaluating the influence of canal curvature on this estimation. Sixty extracted human premolar teeth were selected for this study. Root canal length measurement was performed on CBCT scans (NewTom, Giano, Verona, Italy) and digital paralleling radiography (EzRay Air W; Vatech, Korea). The real working length was established by subtracting 0.5 mm from the actual canal length. No significant difference was found between CBCT and digital paralleling radiography. There was a tendency for underestimation of the root canal length measured on the CBCT images in 52 (86.7%) of the examined teeth and overestimation in 5 teeth (8.3%). All the digital radiographs slightly overestimated the real canal length. The analysis revealed a strong correlation between the estimation from moderate to severe curvature for digital radiography and CBCT images. Preoperative working length estimation can be made closest to its real clinical canal length on the standardized paralleling technique, using a long (16-inch) target-receptor distance.

## 1. Introduction

Radiographic working length estimation is an essential component of the overall endodontic diagnosis and treatment planning process. Conventional 2-dimensional (2D) radiographs provide a cost-effective, high-resolution image, which continues to be the most popular method of imaging today. However, intraoral radiography has some limitations because of its 2-dimensional nature; information may be difficult to interpret, especially in challenging conditions when the anatomy and background pattern are complex [1, 2].

Some drawbacks including distortion, magnification, and superimposition may negatively affect the determination of the accurate working length [3]. Furthermore, periapical radiography fails to provide an accurate location of the apex in cases in which an eccentric apical foramen is present [4]. Radiographic imaging is the most commonly used diagnostic tool in endodontic diagnosis and treatment planning. The image distortions constitute a significant inconvenience. Accuracy of the working length plays a crucial role in determining the success of root canal treatment and could be a predictor of success and possible complications [5]. Overestimation of the endodontic working length may cause overinstrumentation of the root canals, whereas underestimation of the working length may result in insufficient root canal preparation [6, 7].

The accuracy of radiographic methods of length determination also depends on the radiographic technique used. Different studies concluded that tooth length determined by the bisecting angle technique, either correctly or incorrectly angulated, was less accurate than that by the paralleling technique. Even when a parallel technique is used, the elongation of images is approximately 5% [8, 9].

The actual working length is taken to be approximately 0.5 to 1 mm short of the radiographic apex. The radiographic image of the root apex seldom offers a clear view of the terminal part of the canal because of radiographic magnification and distortion effects on its complex intricacy [10]. Magnification can be reduced by keeping the object as close to the film as possible. The distortion can be minimized by positioning the object in the central part of the X-ray beam, using a parallel technique [11]. Measurement of the working length may be much more difficult in curved canals, which makes the measuring difficult [12].

CBCT has been considered useful in endodontics as it can provide 3D images for the detection and visualization of the number and location of roots and canals and identification of unidentified root canals [13, 14]. The additional information provided by CBCT may improve the diagnostic accuracy and confidence in decision-making as well as have an impact on treatment planning [1]. Cone-beam computed tomography overcomes several limitations of conventional radiography. Slices can be selected to avoid adjacent anatomical noise. The spatial relationship of the roots of multirooted teeth can be visualized in three dimensions [15].

Although the overall data on the diagnostic accuracy of periapical radiographs seem to vary among different devices and techniques used, nevertheless, the range of precision in working length estimation based on the degree of canal curvature that is acceptable clinically has not been defined. The primary aim of this in vitro study was to investigate the preoperative working length estimation performed on CBCT scans and digital images using a standardized paralleling technique, in correlation with its real working length as a gold standard. The secondary aim was to examine the influence of root canal curvature on the estimation of root canal length as assessed on CBCT scans and periapical radiographs.

## 2. Materials and Methods

### 2.1. Ethical Approval and Experimental Design

The ethics protocol and the final study proposal for this study were confirmed and accepted (Protocol Number 372/2019). This study has followed the CRIS guidelines (Checklist for Reporting In Vitro Studies) as discussed in the 2014 concept note [16]. This study consisted of 60 extracted human premolar teeth. Fully formed single rooted with varying degrees of curvature was selected after the straight ones eliminated. A tooth with uncommon extreme variations like twisted buccal root or three fused roots was also excluded. There was no information about the patients' age, gender, tooth quadrant, or reason for extraction. Specimens were stored in 10% formalin for a maximum of 2 months. Tissue fragments and calcified debris were removed by using a scaler and then washed under tap water. Finally, they were stored in normal saline at room temperature until use.

### 2.2. Measurements on Digital Intraoral Radiographic Images

Each specimen was embedded in self-curing clear acrylic resin (SP, Brazil) using a cylindrical plastic container. Baseline periapical radiographs with the parallel technique were obtained for all the teeth, to evaluate the tooth anatomy before preparing the access cavity and to estimate radiographic tooth length. Extracoronal metallic restorations were removed. Calcified canals and endodontically treated teeth were also excluded. All the digital periapical radiographs were made by using a high-frequency oral X-ray machine (EzRay Air W; Vatech, Korea), with an exposure time of 0.5 seconds (65 kV, 3.0 mA). To compensate for image magnification, the target-receptor distance was increased to ensure that only the most parallel rays directed toward the tooth and the X-ray sensor. As a result, a long (16-inch) target-receptor distance was used [17]. The images were collected and analyzed. The working length was measured using an open-source image analysis program (EasyDent V4 Viewer, Vatech, Korea).

### 2.3. Measurements on CBCT Images

**Figure 1:**
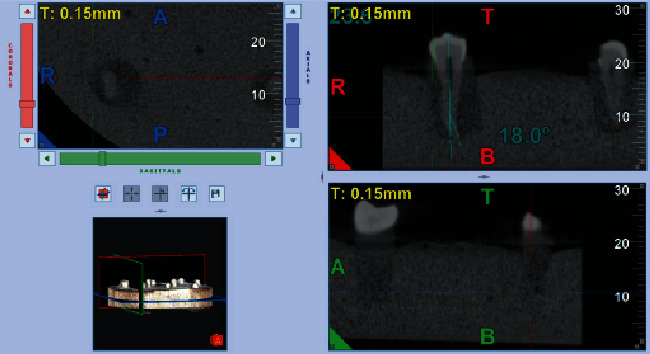
Representative CBCT image; reformatted slice showing the calibration of the root canal pathway.

All CBCT images were obtained and measured by a maxillofacial radiologist not involved in any step of the root canal access preparation. Every ten teeth were arranged inside a custom-made wood box consistently by the aid of dental plaster, after being coated with MS3 master die lubricant separator (Ivoclar Vivadent, USA). The constructed box was to be used as a specific mold for confirming the standardization of the CBCT images. Specimens were scanned from the buccolingual section of each tooth by using CBCT (NewTom Giano, Verona, Italy) with 90 kVp, 3 mA, voxel size (0.3 mm), and FOV 8 cm by 11 cm. Each mold was horizontally fitted to the chin support in a way that the occlusal plane was parallel to the plate [18]. Scan images from the sagittal view were used for the CBCT evaluation, depending on the multiplanar reformatted sections for selecting the best sagittal view for the calibration. The slices were first reformatted to vertically position the root canal of each inspected tooth, to visualize the tooth cusp, pulp chamber, apical foramen, and the complete view of the root canal pathway. All images were converted for viewing with image analysis software (NNT Software, Verona, Italy) to determine canal curvature and estimate radiographic tooth length (Figure 1).

### 2.4. Canal Curvature Measurements

The degree of root canal curvature and the root canal length were measured on both the CBCT scans and digital periapical images. The degree of canal curvature angle was determined by using the Schneider technique, which measures curvature as the acute angle between the long axis of the root canal and a line joining the apical foramen to the point of initial canal curvature [19]. The Schneider method involves first drawing a line parallel to the long axis of the canal, in the coronal third; a second line is then drawn from the apical foramen to intersect the point where the first line left the long axis of the canal. The Schneider angle is the intersection of these lines (Figure 1). The resulted canal angle is named according to the degree of root canal curvature: moderate: 10-25°; severe: 26-70°.

### 2.5. The Estimated and Real Working Length Measurement

**Figure 2:**
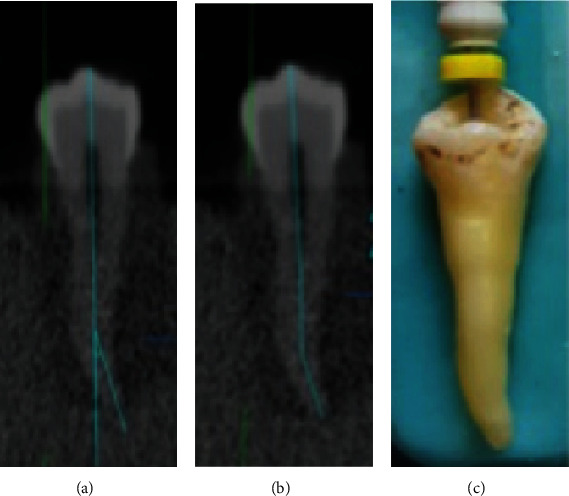
Representative digital image. (a) Canal curvature measurement. (b) Radiographic working length measurements obtained using CBCT imaging. (c) Clinical real length measurement of the same tooth.

The opening with the largest diameter was determined as the major apical foramen. The measurement line was placed in the center of the pulpal cavity and followed each visible canal deviation, thus also allowing for the measurements of nonlinearly shaped canals. The radiographic tooth length was determined on the CBCT and digital periapical images as the distance between the tip of the cusp and the major foramen. The radiographic working length for all the specimens was measured separately on digital periapical and CBCT images after subtraction of 1 mm from the radiographic tooth length. The conventional straight-line access opening was prepared. A standard size #10 or #15 K-File (Dentsply Maillefer, Ballaigues, Switzerland) was passively advanced until its tip was seen at the level of the coronal most boundary of the major apical foramen, by the aid of a magnifying glass (Keeler, Windsor, UK, ×3 magnification). The rubber stopper was set as a reference point and the distance measured with an electronic digital caliper (Mitutoyo Corp., Japan) to the nearest (0.01 mm) and recorded as the actual working length. The real working length was established by subtracting 0.5 mm from the actual canal length and considered as a gold standard in the present study (Figure 2).

### 2.6. Statistical Analysis

The means and standard deviations (SD) were calculated for each group. An independent *t*-test used to compare the mean values of estimated canal length depending on the degree of canal curvature. The Pearson correlation coefficient was calculated based on the data from the CBCT scans and digital radiography to evaluate their accuracy closest to the real length. Tolerance levels in a range of 0.5 mm and 1 mm difference from the real canal length were calculated by the chi-square test. The significance level was set at *P* < 0.05.

## 3. Results

**Figure 3:**
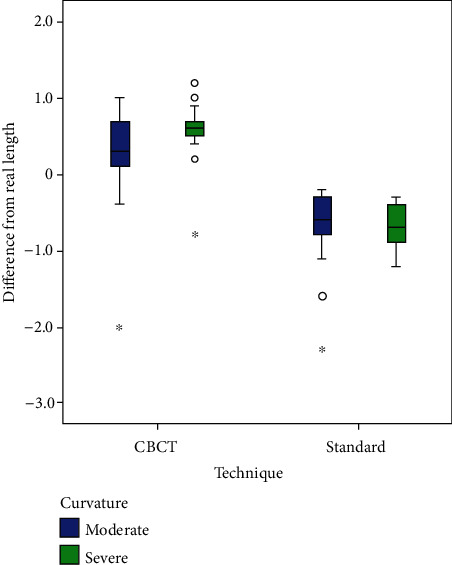
Box plots representing the differences between the estimated working length measurements obtained by using CBCT and digital paralleling technique, closest to the real clinical values (a positive value means overestimation, and a negative value means underestimation).

A comparison of mean (standard deviation) values of estimated canal length assessed using three methods depending on the degree of canal curvature is summarized in (Tables 1 and 2). There was no significant effect of the degree of canal curvature on the accuracy of each of the methods (*P* > 0.05). There was a tendency for underestimation of the root canal length measured on the CBCT images in comparison with the real root canal length from -1.2 mm to -0.1 mm (−0.538 ± 0.303) in 52 (86.7%) of the examined teeth and overestimation from 0.1 mm to 2.0 mm (0.7 ± 0.775) in 5 teeth (8.3%). All the digital paralleling radiographic images slightly overestimated the real canal length mean value (0.635 ± 0.374). Despite this overestimation, this method showed an overall accuracy of 53.3% (32) and 88.3% (51) within the range of +0.5 mm and +1 mm tolerance levels, respectively (Table 2). Of the 60 teeth scanned, the CBCT images showed an accuracy of 55% (33) and 96.7% (58) within a range of +0.5 mm and +1 mm, respectively. There were no statistically significant differences between the digital paralleling technique and CBCT measurements within a range of 0.5 mm and 1 mm tolerance levels from the gold standard (*P* > 0.05) (Tables 3 and 4). The distributions of the differences in the estimated working length values obtained using CBCT and digital paralleling technique closest to the real clinical values are presented in (Figure 3).

**Figure 4:**
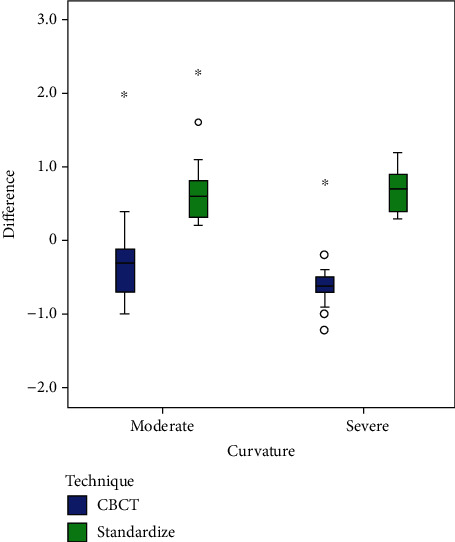
Box plots comparing the impact of the degree of root canal curvatures in determining the estimated working length by digital paralleling radiography and CBCT technique.

The Pearson correlation coefficients for comparing the degree of root canal curvature in determining the working length by digital paralleling radiography and CBCT technique showed a strong correlation between the measurements from moderate to severe canal curvature for digital radiography (*r* = 0.972 to 0.938, *P* < 0.001) and CBCT images (0.952 to 0.912, *P* < 0.001) (Table 5 and Figure 4).

## 4. Discussion

This study has twofold objectives. The first is to draw inferences from the collected objective findings from the present investigations and existing/known information confirmed in the scientific literature. The measurements were obtained with the two imaging techniques, CBCT and digital periapical radiography, closest to their real canal length, and assessed their diagnostic validity and the possible influence of other associated factors such as degree of canal curvature. The second objective is to allow an explanation for the observed differences and alikeness, with relevant studies that used similar methodology (CBCT imaging) to obtain an evidence-based background, which might be valuable in clinical practice.

Several techniques are available for the estimation of diagnostic working length. Some protocols are more suitable for clinical use, and others are designed well as a research tool. Factors such as case difficulty and the technique sensitivity, as well as access to the various imaging techniques and imaging software, are likely to influence the choice of technique to estimate root canal length.

In this in vitro study, the correlation between the accuracy of root canal length estimation and its degree of canal curvature in human premolar teeth was investigated under *ex vivo* conditions. By using extracted teeth and by applying standard protocols, it is possible to establish a “real” root canal length through direct clinical examination of the apical foramen. It can be used as a gold standard since the reliability, accuracy, and reproducibility of this method had previously been validated in the literature [3].

The real clinical working length was established by subtracting 0.5 mm from the actual canal length (file tip at the level of the coronal most boundary of the major foramen) and considered as a gold standard in the present study. Radiographic working length was measured separately using digital periapical radiographs and CBCT images. The final radiographic working length was calibrated by subtracting 1 mm from the radiographic tooth length since the ideal apical limit for all intracanal procedures is the narrowest point of the canal, the so-called apical constriction or the dentinocemental junction. This point is located 0.5 to 1.0 mm short of the radiographic apex of the root [20, 21].

The results of the present experiment found clear support for the standardized paralleling angle technique, since it is accurate and showed no significant differences in comparison with the CBCT measurement and real clinical working length value (gold standard). It is easier to do standardization for all the radiographs clinically, which allows the positioning of the film and the object in approximately the same position. However, some limitations of our implementation need to be considered, such as two-dimensional magnification, factors related to the direction of root angulation, anatomic variations, and location of the major foramen. The inspection of the third dimension of the root canal helps to increase the accuracy of working length estimation. Therefore, measurements of root canals on existing CBCT images are a relatively new method for the confirmation of the diagnostic working length, which has been studied by numerous researchers [22, 23]. However, the CBCT scan is not recommended to be used routinely in endodontic practice because of dose considerations and potential radiation risks [24, 25].

In this study, neither of the digital radiography systems and CBCT showed a significant difference with the gold standard. However, the digital radiography revealed to be less accurate than CBCT imaging in correlation with the gold standard, which can be related to the dimensional distortion of the image, and the root canals frequently did not end close to the radiographical apical reference point. This anatomical variation might result in an overestimation of the canal length measured by the radiographic method. In line with these common findings, with that of several previous studies, the lower accuracy in root canal length estimation from periapical radiograph than from CBCT scans is confirmed [26, 27].

The present study confirmed the accuracy of the canal length measurements performed with the use of radiographic images, in various degrees of curvature. In line with previous studies, both CBCT and digital working length measurements were slightly more accurate when performed in canals having a moderate canal curvature, although there was no statistically significant difference in mean values of root canal length with a severe curvature angle [28]. These results explain the fact that the exact direction and nature of root angulation and anatomic noise result in limited information on the root canal anatomy of the tooth assessed with the 2-dimensional conventional radiography. CBCT imaging overcomes this by allowing the multiplanar evaluation, which leads to a clear visualization of the root canal configuration and canal curvature.

It is crucial to highlight the fact that the results of this study are to be considered specific to the radiographic technique applied to compensate for image magnification. As a result, a long (16-inch) target-receptor distance was used. This compensation is a significant point for the broad implication of the present research in clinical practice. Although paralleling techniques produce less projection and procedural errors than bisecting angle technique, ideal film and X-ray tube head orientation can be difficult to achieve in some clinical situations because it can be complicated by anatomic variations [17]. On the other side, CBCT scanning evaluated, in the absence of streak and beam hardening artifacts, photon starvation, caused by metal restorations, brackets, implants, and metal root fillings; motion artifacts; and anatomic noise from the opposing jaw structures. Isolating artifacts three-dimensionally has been a challenge in clinical endodontics.

## 5. Conclusions

The conclusion drawn is that a standardized parallel radiographic method was performed similarly to the CBCT technique and closest to its real clinical working length using a long (16-inch) target-receptor distance. It could prevent the need for further diagnostic radiographs for confirmation of working canal length.

## Figures and Tables

**Table 1:** Comparison of mean (standard deviation) values of estimated canal length assessed using two methods depending on the degree of canal curvature.

		Mean ± SD		Total	*P* value^∗^
		Curvature			
		Moderate	Severe	(%)	
Overestimate paralleling technique	Estimated length	20.86 ± 1.63	20.43 ± 0.83	20.72 ± 1.43	0.27^∗∗^
	Difference from real length	0.624 ± 0.409	0.658 ± 0.293	0.635 ± 0.374	0.75^∗∗^
	Min to max of difference	(0.2 to 2.3)	(0.3 to 1.2)	(0.2 to 2.3)	
	{*N*}	{*n* = 41}	{*n* = 19}	{*n* = 60, 100%}	

Overestimate CBCT	Estimated length	19.43 ± 1.22	20.3±	19.6 ± 1.13	0.47^∗∗^
	Difference from real length	0.675 ± 0.892	0.80±	0.7 ± 0.775	0.91^∗∗^
	Min to max of difference	(0.1 to 2.0)	-0.8	(0.1 to 2.0)	
	{*N*}	{*n* = 4}	{*n* = 1}	{*n* = 5, 8.3%}	

Underestimate CBCT	Estimated length	19.83 ± 1.72	19.41 ± 1.02	19.70 ± 1.54	0.81^∗∗^
	Difference from real length	−0.494 ± 0.306	−0.638 ± 0.280	−0.538 ± 0.303	0.12^∗∗^
	Min to max of difference	(-1.0 to -0.1)	(-1.2 to -0.2)	(-1.2 to -0.1)	
	{*N*}	{*n* = 36}	{*n* = 16}	{*n* = 52, 86.7%}	

^∗^Independent *t*-test. ^∗∗^Not significant.

**Table 2:** Measurement reproducibility in digital paralleling radiography and CBCT scan closest to the real values, in a range of 0.5 mm and 1 mm.

		Curvature	Total	*P* value^∗^	
Accurate estimation		Moderate	Severe		
CBCT (0.5 mm)	Yes	26 (60.5%)	7 (41.2%)	33 (55%)	0.18^∗∗^
	No	17 (39.5%)	10 (58.8%)	27 (45%)	

CBCT (1 mm)	Yes	42 (97.7%)	16 (94.1%)	58 (96.7%)	0.49^∗∗^
	No	1 (2.3%)	1 (5.9%)	2 (3.3%)	

Paralleling technique (0.5 mm)	Yes	25 (48.8%)	7 (41.18%)	32 (53.3%)	0.92^∗∗^
	No	18 (51.2%)	10 (58.82%)	28 (46.7%)	

Paralleling technique (1 mm)	Yes	36 (83.72%)	15 (88.2%)	51 (85%)	0.85^∗∗^
	No	7 (16.28%)	2 (11.8%)	9 (15%)	

^∗^Chi-square test. ^∗∗^Not significant.

**Table 3:** Comparison between the measurements obtained by digital paralleling and CBCT techniques within a range of 0.5 mm difference from the gold standard.

		Accuracy of digital paralleling technique by 0.5 mm		Total	*P* value^∗^
		Yes	No		
Accuracy of CBCT by 0.5 mm	Yes	17	16	33	0.59^∗∗^
	No	12	15	27	
Total		29	31	60	

^∗^Chi-square test. ^∗∗^Not significant.

**Table 4:** Comparison between the measurements obtained by digital paralleling and CBCT techniques within a range of 1 mm difference from the gold standard.

		Accuracy of digital paralleling technique by 1 mm		Total	*P* value^∗^
		Yes	No		
Accuracy of CBCT by 1 mm	Yes	52	6	58	0.09^∗∗^
	No	1	1	2	
Total		53	7	60	

^∗^Chi-square test. ^∗∗^Not significant.

**Table 5:** Regression analysis to show linear correlation between the real length and estimated length in both techniques (digital radiography and CBCT) with different root curvatures.

		Real working length		*P* value
		*R*	*R* ^2^	
Digital paralleling technique	Curvature = moderate	0.972	0.944	<0.001
	Curvature = severe	0.938	0.88	<0.001
CBCT	Curvature = moderate	0.952	0.906	<0.001
	Curvature = severe	0.912	0.831	<0.001

*R* and *R*^2^ performed by linear regression analysis.

## Data Availability

The numerical data used to support the findings of this study are available from the corresponding author upon request.
